# The Obesity Mortality Paradox in Patients with Pulmonary Embolism: Insights from a Tertiary Care Center

**DOI:** 10.3390/jcm13082375

**Published:** 2024-04-19

**Authors:** Fahad Alkhalfan, Syed Bukhari, Akiva Rosenzveig, Rohitha Moudgal, Syed Zamrak Khan, Mohamed Ghoweba, Pulkit Chaudhury, Scott J. Cameron, Leben Tefera

**Affiliations:** 1 Department of Cardiovascular Medicine, Section of Vascular Medicine, Heart Vascular and Thoracic Institute, Cleveland Clinic Foundation, Cleveland, OH 44195, USA; alkhalf2@ccf.org (F.A.); bukhars6@ccf.org (S.B.); rosenza@ccf.org (A.R.); moudgar@ccf.org (R.M.); khans15@ccf.org (S.Z.K.); ghowebm@ccf.org (M.G.); chaudhp3@ccf.org (P.C.); cameros3@ccf.org (S.J.C.); 2 Department of Cardiovascular and Metabolic Sciences, Lerner Research Institute, Case Western Reserve University, Cleveland, OH 44120, USA; 3 Department of Hematology, Taussig Cancer Institute, Cleveland Clinic Foundation, Cleveland, OH 44195, USA

**Keywords:** pulmonary embolism, obesity, mortality, pulmonary embolism response team

## Abstract

**Background:** While obesity is associated with an increased risk of venous thromboembolism (VTE), there is some data to suggest that higher BMI is also associated with decreased all-cause mortality in patients with a pulmonary embolism (PE). **Methods:** Using PE Response Team (PERT) activation data from a large tertiary hospital between 27 October 2020 and 28 August 2023, we constructed a multivariate Cox proportional hazards model to assess the association between obesity as a dichotomous variable (defined as BMI ≥ 30 vs. BMI 18.5–29.9), BMI as a continuous variable, and 30-day PE-related mortality. **Results:** A total of 248 patients were included in this analysis (150 with obesity and 98 who were in the normal/overweight category). Obesity was associated with a lower risk of 30-day PE-related mortality (adjusted HR 0.29, *p* = 0.036, 95% CI 0.09–0.92). A higher BMI was paradoxically associated with a lower risk of PE-related mortality (HR = 0.91 per 1 kg/m^2^ increase, *p* = 0.049, 95% CI 0.83–0.999). **Conclusions:** In our contemporary cohort of patients with a PERT activation, obesity was associated with a lower risk of PE-related mortality.

## 1. Introduction

Pulmonary embolism (PE) is the third most common cause of cardiovascular death in the United States [1] with an estimated mortality approaching 300,000 annually [2]. The advent of the multidisciplinary pulmonary embolism response team (PERT) and novel minimally invasive catheter-based treatments has led to a reduction in mortality [3,4,5]. Yet, despite the improvements gained, there is a pressing need to identify patients who are at a higher risk of morbidity and mortality.

The prevalence of obesity has been steadily rising, with recent data indicating that 42% of US adults are obese [6]. Obesity is shown to be a significant risk factor of venous thromboembolism (VTE) [7]. A prospective analysis of the Nurses’ Health study demonstrated a profound linear association between body mass index (BMI) and the risk of PE, even with modest increases in BMI [8]. The obesity paradox is the concept that despite being a risk factor for various disease states, obesity portends improved outcomes. Although initially described in hemodialysis patients in 1999 [9], it has subsequently been shown in coronary artery disease [10], heart failure [11], and chronic obstructive pulmonary disease (COPD) [12]. A retrospective analysis of PE using the RIETE registry showed a potential signal for reduced all-cause mortality in patients who were overweight [13]. More recently, Keller et al. demonstrated decreased all-cause mortality in class I and II obesity but class III obesity had similar outcomes with non-obese patients [14]. While quite informative, studies to date have been unable to ascertain rates of PE-specific mortality in patients with obesity. In addition, previous studies were performed prior to the era of catheter-based therapies.

Thus, this study sought to identify differences in demographic and treatment characteristics between obese and non-obese patients and to further elucidate the association of obesity with mortality, particularly PE-specific mortality.

## 2. Methods

This was an analysis of all patients with a PERT activation between 27 October 2020 and 28 August 2023 in a large tertiary care center. Clinical data, including demographics, co-morbidities, hospital course, and relevant outcomes, including mortality, were ascertained from patients’ electronic medical records. Patients who were classified as having a low risk of mortality according to the European Society of Cardiology (ESC) were excluded from this analysis as the PE in this situation is not significant enough to cause hemodynamic compromise and unlikely to be contributing to PE mortality.

The primary aim of this analysis was to determine if obesity as a dichotomous variable (BMI of 30 or greater vs. 18.5 to 29.9) was associated with a decreased risk of 30-day PE-related mortality. Patients with a BMI of less than 18.5 were excluded from the study as we did not want to assume that they would have similar event rates as compared to the BMI of 18.5–30 cohort. A death was considered related to the PE if it was believed to occur as a direct complication of the PE (such as worsening hypoxia, cardiogenic shock, or cardiac arrest) or as a result of subsequent treatment (such as anticoagulation leading to a fatal major bleeding event such as intracranial hemorrhage). If the cause of death was unknown or uncertain, it was categorized as not being related to PE. Secondary outcomes included 30-day all-cause mortality, in-hospital major bleeding, and the length of admission. The association between BMI, as a continuous variable, and the previously mentioned outcomes was also assessed.

### 2.1. Statistical Analysis

Baseline variables were compared between patients who were obese and those who were either in the non-obese or overweight BMI range. Differences in characteristics between the two groups were compared using a *t*-test or Wilcoxon rank sum for continuous variables depending on the distribution of the values and a Chi-square test for categorical values.

To identify variables that would be included in the final model, we utilized a backward selection approach to identify variables that would be included in our final multivariable Cox proportional hazards model. A *p*-value of 0.40 was designated as the cut off to exclude variables. This cut off was selected to maximize the number of variables included in the model. We included baseline demographic and clinical variables as potential parameters. This included sex (male vs. female), age, race, ESC PE mortality risk, smoking status (active smoker vs. non-smoker), history of cancer, history of heart disease, history of chronic lung disease, prior VTE, recent COVID infection (a positive test within the last 30 days or history of COVID within the last 30 days), and hypotension (systolic BP < 90 mm Hg). We also re-ran the same model with BMI as a continuous variable.

As for the secondary outcomes of interest, we utilized a multivariable Cox proportional hazards model to assess the association between obesity and all-cause 30-day mortality. We adjusted for the same variables used for our primary outcome of interest. We utilized the Fisher’s exact test to assess the association between obesity and major bleeding (all major bleeding, extracranial major bleeding, and intracranial major bleeding separately). We utilized the International Society on Thrombosis and Hemostasis (ISTH) criteria for major bleeding (Fatal bleeding, symptomatic bleeding in a critical organ or a fall in hemoglobin by more than 2 g/dL, or transfusion of at least 2 units of packed red blood cells) [15]. We assessed the difference in the length of hospitalization between the two groups using the Wilcoxon rank sum test. A *p*-value of <0.05 was determined to be statistically significant. We did not adjust for multiple comparisons. The analysis was conducted using STATA MP/16.1 (College Station, TX, USA).

### 2.2. Sensitivity Analysis

To account for the differences between obese and non-obese patients that were not included in our final multivariable Cox proportional hazards model, we included additional variables that were significantly different between the two groups (*p* < 0.05). We also included advanced PE treatments if they had not already been included in the final model. We re-ran two versions of the model: obesity as a dichotomous variable as described previously and BMI as a continuous variable.

## 3. Results

A total of 248 patients were included in this analysis (150 patients with a BMI ≥ 30 and 98 patients with a BMI between 18.5 and 29.9). A comparison of baseline demographics can be seen in Table 1. To summarize, patients who were obese tended to be younger (mean age 59.9 years vs. 65.5, *p* = 0.005), were less likely to have a history of cancer (22.0% vs. 39.8%, *p* = 0.003), and a higher proportion of patients had a recent COVID infection (13.3% vs. 4.1%, *p* = 0.016). There was no significant difference in sex, history of prior VTE, history of heart failure or chronic lung disease, or hypotension at presentation. Additionally, the distribution of intermediate–low-, intermediate–high-, and high-risk patients was similar between the two groups. Finally, the rates of advanced treatments, including catheter-based thrombectomy and systemic thrombolysis, were similar between patients who were obese and those who were non-obese or overweight.

**Figure 1:**
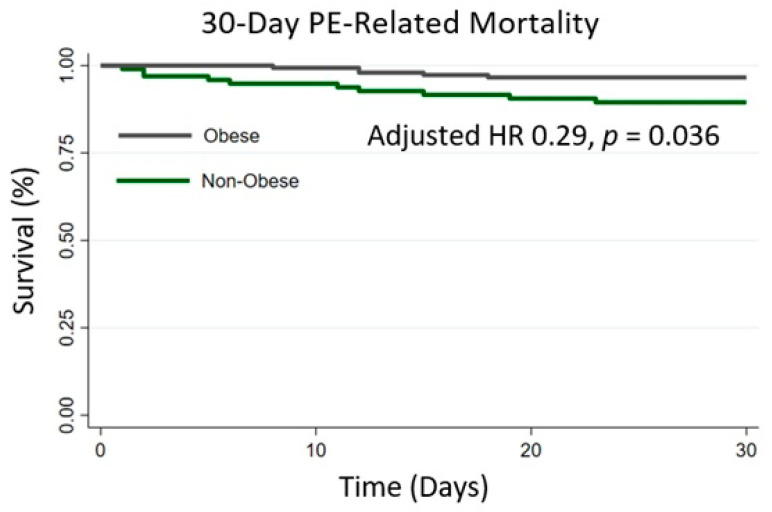
Kaplan–Meier curve for 30-day PE-related mortality.

There were 24 deaths from all causes at 30 days (7 (4.8%) in the obesity group and 17 (17.4%) in the non-obese/overweight group). Of those, 15 deaths were determined to be because of a complication of PE or its subsequent treatment (5 in the obesity group (3.4%) and 10 (10.5%) in the normal/overweight group). As seen in Figure 1, the separation occurred relatively early and persisted during the entire follow-up period. In an unadjusted analysis, obesity (as compared to patients in the non-obese/overweight category) was associated with a lower risk of 30-day PE-related mortality (Hazard ratio (HR) 0.31, *p* = 0.031, 95% CI 0.10–0.90) and all-cause mortality (HR 0.25, *p* = 0.002, 95% CI 0.10–0.61). This association remained significant when BMI was treated as a continuous variable ((PE-related mortality: 0.90, *p* = 0.023, 95% CI 0.82–0.99); (all-cause mortality: 0.91, *p* = 0.007, 95% CI 0.85–0.97)).

As seen in Table 2, the final multivariable Cox proportional hazards model adjusted for sex, age, history of heart failure, history of chronic lung disease, recent COVID infection, use of systemic thrombolysis, and ESC PE mortality risk. After adjustment, the association between obesity and 30-day PE-related death remained significant (HR 0.29, *p* = 0.036, 95% CI 0.09–0.92). This association remained significant when BMI was treated as a continuous variable (HR 0.91, *p* = 0.049, 95% CI 0.83–0.999) (Table 3). As seen in Supplemental Tables S1 and S2, this pattern remained consistent when assessing for 30-day all-cause mortality (obesity: adjusted HR: 0.25, *p* = 0.004, 95% CI 0.10–0.64; BMI: adjusted HR 0.92, *p* = 0.018, 95% CI 0.86–0.99). There was no statistically significant difference in the duration of hospitalization (obesity: median 9 days, IQR 6–18; non-obese/overweight: median 9 days, IQR 6–17; *p* = 0.66).

The sensitivity analysis included history of cancer and catheter-related thrombectomy as additional parameters. The association between obesity as a dichotomous variable and PE-related mortality remained statistically significant (HR 0.29, *p* = 0.039, 95% CI 0.09–0.94). However, the association between BMI as a continuous outcome and PE-related death was no longer significant (HR 0.91, *p* = 0.063, 95% CI 0.83–1.01).

As for our other secondary outcomes of interest, the overall rate of major bleeding was lower in the obesity group, but this did not reach statistical significance (six (4.1%) vs. eight (8.8%), *p* = 0.16). However, when stratified by extracranial vs. intracranial major bleeding, there was a significantly lower rate of extracranial major bleeding in the obese group (1 (0.7%) vs. 8 (8.8%), *p* = 0.002) with no statistically significant difference in the rate of intracranial bleeding (4 (2.7%) vs. 0 (0.0%), *p* = 0.30).

## 4. Discussion

Our study found that obesity was associated with a lower risk of PE-related mortality and all-cause mortality in PERT patients who had an intermediate or high risk of mortality. Additionally, overall major bleeding rates and the length of hospitalization was similar between obese and non-obese individuals. However, obesity was associated with lower rates of extracranial major bleeding.

Obesity has a well-known association with increased risk of PE, owing in part to increased platelet reactivity [16]. However, paradoxically, patients with obesity have better survival rates with PE compared to non-obese patients—a phenomenon known as obesity paradox. Keller et al. studied the German national database of >345,000 patients with acute PE and demonstrated that obesity was associated with lower all-cause in-hospital mortality rate regardless of age, sex, comorbidities, and reperfusion treatment [14]. Stein et al. used Nationwide Inpatient Sample to demonstrate that all-cause mortality in patients with PE is lower in patients with obesity than in non-obese/under-weight patients, with the greatest effects seen in women and older patients [17].

While the exact mechanism is unknown and it is unclear whether obesity paradox is related to protective effect of increased body fat, few pathophysiologic hypotheses regarding the “obesity paradox” in PE have been proposed. Patients with obesity tend to have greater right ventricular (RV) mass and higher RV volume compared with non-obese, which could potentiate their ability to cope with acute increases in RV overload [18]. Patients with obesity also have greater left ventricular (LV) mass and thicker interventricular septum that can make them more resistant to septal bowing, and potentially mitigate the risk of developing obstructive shock [19]. Some argue that obesity paradox does not necessarily reflect the protective effect of obesity but instead reflects the case of metabolically healthy obese individuals, a frequent and common finding in modern societies. These individuals have a better metabolic reserve, better smoking profile, and/or less disease-associated weight loss [20]. Non-PE-based studies have highlighted that cardiopulmonary fitness is an often-overlooked potential modifier of obesity paradox, and levels of fitness can significantly alter the inverse association between obesity and mortality [21]. The alternative explanation may be that BMI is not be the ideal definition of obesity [22]. In a study from the UK Biobank, waist–hip ratio was found to have the strongest association with all-cause mortality when compared with BMI and fat mass index [23].

Our study demonstrates some important findings that have not been reported previously. Firstly, unlike other studies that have reported all-cause mortality as the primary outcome while evaluating the impact of obesity in PE patients, we investigated PE-related deaths as the primary outcome. This helped us to mitigate the influence of confounders on our study analysis such as malignancy, congestive heart failure, etc. that are common non-PE-related causes of death in PE patients. Secondly, our patient population comprised intermediate to high-risk subjects from PERT registry, signifying sicker population with greater disease burden compared with previously reported cohorts. Thirdly, a higher utilization of catheter thrombectomy in our study population makes our study more compatible with the contemporary real-world practices and gives more credence to our results. As seen in Table 1, there was no statistically significant difference in the rates of advanced treatments, including catheter thrombectomy or systemic thrombolysis, between the two groups. Also, we conducted a sensitivity analysis to adjust for variables that were not chosen as part of our backward selection method but were still believed to be clinically significant, such as age and catheter thrombectomy. Finally, ~10% of our patients had a recent COVID infection, making them more vulnerable to thrombotic events. Earlier studies have indicated that obesity paradox is not applicable in patients with COVID, as patients with COVID and obesity exhibit more severe disease, are admitted to the intensive care unit more frequently, and have a higher mortality [7,8]. In our study, the obese group had significantly larger number of patients with recent COVID infection compared to the non-obese group, and yet had lower mortality, potentially signaling that obesity paradox may be applicable in patients with COVID and PE. However, we did adjust for COVID infection in our models.

Our study has some limitations. Firstly, being a single-center study, it lacks external generalizability. Additionally, considering the observational nature of this study, association does not imply causation. We attempted to mitigate this bias by performing multivariable analyses adjusting for difference in baseline characteristics; however, the presence of other confounders that were not accounted for could not be excluded (such as diabetes). Also, as we were only looking at PE-related mortality, the event rate was relatively low, and we were likely overfitting the model. However, we were still able to show that obesity was associated with a statistically significant reduction in PE mortality in both the univariate and multivariable models. We also did not adjust for multiple comparisons. Finally, baseline functional status of these patients could not be accurately assessed, which itself is a predictor of mortality. There remains a need for larger, multicentered studies to better evaluate the impact of obesity on morbidity and mortality in PE patients.

## 5. Conclusions

In our cohort of contemporary patients who had a PERT activation and an intermediate or high mortality risk, obesity was associated with a significant reduction in PE-related and all-cause mortality. Additionally, there was no statistically significant difference in major bleeding rates and the length of admission between obese individuals and those in the non-obese/overweight category.

## Figures and Tables

**Table 1:** Baseline Demographics.

Variable		BMI ≥ 30 (*N* = 150)	BMI 18.5–29.9 (*N* = 98)	*p*-Value
Male		70 (46.7%)	55 (56.1%)	0.15
Age, mean (SD)		59.9 (15.3)	65.5 (15.2)	0.005
Prior VTE		7 (4.7%)	4 (4.1%)	0.83
Recent COVID infection		20 (13.3%)	4 (4.1%)	0.016
History of cancer		33 (22.0%)	39 (39.8%)	0.003
History of heart failure		26 (17.3%)	15 (15.3%)	0.67
History of chronic lung disease		29 (19.3%)	24 (24.5%)	0.33
Smoking		19 (12.7%)	15 (15.8%)	0.49
Hypoxia (defined as requiring oxygen)		116 (77.3%)	76 (78.4%)	0.85
Hypotension		13 (8.7%)	13 (13.4%)	0.24
European Society of Cardiology PE Mortality Risk	Intermediate–Low	30 (20.0%)	27 (27.6%)	0.38
	Intermediate–High	85 (56.7%)	51 (52.0%)	
	High	35 (23.3%)	20 (20.4%)	
Catheter thrombectomy		80 (53.7%)	46 (46.9%)	0.30
Systemic thrombolysis		13 (8.7%)	8 (8.2%)	0.89

**Table 2:** Final multivariable Cox proportional hazards model for the association between obesity and 30-Day PE-related mortality.

	Hazard Ratio	*p*-Value	95% Confidence Interval
Obesity (BMI ≥ 30 vs. BMI 18.5–29.9)	0.29	0.04	0.09–0.92
Male	0.54	0.27	0.18–1.60
Age (per 1 year)	1.02	0.27	0.98–1.06
History of Heart Failure	1.98	0.23	0.65–6.04
History of Chronic Lung Disease	2.09	0.18	0.72–6.05
Recent COVID Infection	2.56	0.25	0.52–12.6
Systemic Thrombolysis	3.14	0.12	0.76–13.0
ESC Mortality Risk (Baseline: Intermediate–Low Risk)			
Intermediate–High Risk	0.29	0.06	0.08–1.06
High Risk	0.61	0.46	0.17–2.22

**Table 3:** Final multivariable Cox proportional hazards model for the association between body mass index and 30-day PE-related mortality.

	Hazard Ratio	*p*-Value	95% Confidence Interval
BMI (per 1 kg/m^2^)	0.91	0.049	0.83–0.999
Male	0.53	0.30	0.16–1.74
Age (per 1 year)	1.01	0.47	0.98–1.05
History of Heart Failure	2.40	0.14	0.75–7.72
History of Chronic Lung Disease	2.04	0.23	0.64–6.50
Recent COVID Infection	2.58	0.25	0.52–12.9
Systemic Thrombolysis	3.09	0.12	0.75–12.7
ESC Mortality Risk (Baseline: Intermediate–Low Risk)			
Intermediate–High Risk	0.43	0.22	0.11–1.66
High Risk	0.64	0.53	0.16–2.59

## Data Availability

The data presented in this study are available on request from the corresponding author. The data are not publicly available due to institutional policy.

## Supplementary Materials

The following supporting information can be downloaded at: https://www.mdpi.com/article/10.3390/jcm13082375/s1, Table S1: Multivariable Cox-Proportional Hazards Model for the Association Between Obesity and 30-Day All-Cause Mortality, Table S2: Multivariable Cox-Proportional Hazards Model for the Association Between BMI and 30-Day All-Cause Mortality, Table S3: Sensitivity Analysis: Multivariable Cox-Proportional Hazards Model for the Association Between Obesity and 30-Day PE-Related Mortality, Table S4: Sensitivity Analysis: Multivariable Cox-Proportional Hazards Model for the Association between BMI and 30-Day PE-Related Mortality.
