# Supplementary material for: The Obesity Mortality Paradox in Patients with Pulmonary Embolism: Insights from a Tertiary Care Center
Source: J Clin Med. 2024 Apr 19;13(8):2375. doi: 10.3390/jcm13082375 (PMC11051153; doi:10.3390/jcm13082375)
Supplement: Supplementary file 1 [file jcm-13-02375-s001.zip › jcm-2938819-supplementary.pdf]

Supplemental Table S1. Multivariable Cox-Proportional Hazards Model for the Association Between Obesity and 30-Day All-Cause Mortality

	Hazard Ratio	P-value	95% Confidence Interval
Obesity (BMI >= 30 vs BMI 18.5 – 29.9)	0.25	0.004	0.10 – 0.64
Male	0.69	0.38	0.30 – 1.57
Age (per 1 year)	1.01	0.56	0.98 – 1.04
History of Heart Failure	1.60	0.34	0.61 – 4.17
History of Chronic Lung Disease	1.49	0.37	0.62 – 3.56
Recent COVID Infection	1.52	0.59	0.34 – 6.87
Systemic Thrombolysis	1.69	0.45	0.44 – 6.54
ESC Mortality Risk (Baseline: Intermediate-Low Risk)			
Intermediate-High Risk	0.30	0.02	0.11 – 0.79
High Risk	0.63	0.40	0.22 – 1.84

Supplemental Table S2. Multivariable Cox-Proportional Hazards Model for the Association Between BMI and 30-Day All-Cause Mortality

	Hazard Ratio	P-value	95% Confidence Interval
BMI (per 1 kg/m^2 increase)	0.92	0.02	0.86 - 0.99
Male	0.76	0.52	0.32 - 1.77
Age (per 1 year)	1.01	0.68	0.98 - 1.03
History of Heart Failure	1.72	0.27	0.65 - 4.56
History of Chronic Lung Disease	1.52	0.37	0.61 - 3.80
Recent COVID Infection	1.32	0.72	0.30 - 5.90
Systemic Thrombolysis	1.63	0.48	0.43 - 6.23
ESC Mortality Risk (Baseline: Intermediate-Low Risk)			
Intermediate-High Risk	0.37	0.054	0.14 - 1.02
High Risk	0.63	0.41	0.20 - 1.92

Supplemental Table S3. Sensitivity Analysis: Multivariable Cox-Proportional Hazards Model for the Association Between Obesity and 30-Day PE-Related Mortality

	Hazard Ratio	P-value	95% Confidence Interval
Obesity	0.29	0.04	0.09 - 0.94
Male	0.55	0.28	0.18 - 1.64
Age (per 1 year)	1.02	0.28	0.98 - 1.06
Systemic thrombolysis	3.10	0.13	0.73 - 13.18
History of heart failure	1.92	0.27	0.60 - 6.11
History of chronic lung disease	2.04	0.19	0.70 - 5.95
History of cancer	1.15	0.82	0.36 - 3.71
COVID Infection	2.75	0.23	0.52 - 14.38
Catheter thrombectomy	1.19	0.76	0.39 - 3.64
ESC Mortality Risk (Baseline: Intermediate-Low Risk)			
Intermediate-High Risk	0.29	0.07	0.08 - 1.09
High Risk	0.62	0.47	0.17 - 2.24

Supplemental Table S4. Sensitivity Analysis: Multivariable Cox-Proportional Hazards Model for the Association Between BMI and 30-Day PE-Related Mortality

	Hazard Ratio	P-value	95% Confidence Interval
BMI (per 1 kg/m^2)	0.91	0.06	0.83 - 1.01
Male	0.56	0.34	0.17 - 1.83
Age (per 1 year)	1.01	0.46	0.98 - 1.06
Systemic Thrombolysis	2.85	0.16	0.67 - 12.15
History of Heart Failure	2.31	0.18	0.67 - 7.92
History of Chronic Lung Disease	1.87	0.30	0.57 - 6.18
Recent COVID Infection	3.18	0.18	0.58 - 17.39
Catheter Thrombectomy	1.42	0.57	0.42 - 4.81
History of cancer	1.53	0.53	0.41 - 5.66
ESC Mortality Risk (Baseline: Intermediate-Low Risk)			
Intermediate-High Risk	0.44	0.25	0.11 - 1.79
High Risk	0.69	0.61	0.17 - 2.81
